# The importance of copy number variation in congenital heart disease

**DOI:** 10.1038/npjgenmed.2016.31

**Published:** 2016-09-14

**Authors:** Gregory Costain, Candice K Silversides, Anne S Bassett

**Affiliations:** 1 Clinical Genetics Research Program, Centre for Addiction and Mental Health, Toronto, ON, Canada; 2 Medical Genetics Residency Training Program, University of Toronto and Division of Clinical and Metabolic Genetics, The Hospital for Sick Children, Toronto, ON, Canada; 3 The Toronto Congenital Cardiac Centre for Adults, Division of Cardiology, University Health Network and Mount Sinai Hospital, Toronto, ON, Canada; 4 Department of Psychiatry, Toronto General Research Institute and The Dalglish Family Hearts and Minds Clinic for 22q11.2 Deletion Syndrome, University Health Network, Toronto, ON, Canada; 5 Department of Psychiatry, University of Toronto, and Campbell Family Mental Health Research Institute, Centre for Addiction and Mental Health, Toronto, ON, Canada

## Abstract

Congenital heart disease (CHD) is the most common class of major malformations in humans. The historical association with large chromosomal abnormalities foreshadowed the role of submicroscopic rare copy number variations (CNVs) as important genetic causes of CHD. Recent studies have provided robust evidence for these structural variants as genome-wide contributors to all forms of CHD, including CHD that appears isolated without extra-cardiac features. Overall, a CNV-related molecular diagnosis can be made in up to one in eight patients with CHD. These include *de novo* and inherited variants at established (chromosome 22q11.2), emerging (chromosome 1q21.1), and novel loci across the genome. Variable expression of rare CNVs provides support for the notion of a genetic spectrum of CHD that crosses traditional anatomic classification boundaries. Clinical genetic testing using genome-wide technologies (e.g., chromosomal microarray analysis) is increasingly employed in prenatal, paediatric and adult settings. CNV discoveries in CHD have translated to changes to clinical management, prognostication and genetic counselling. The convergence of findings at individual gene and at pathway levels is shedding light on the mechanisms that govern human cardiac morphogenesis. These clinical and research advances are helping to inform whole-genome sequencing, the next logical step in delineating the genetic architecture of CHD.

Genome-wide rare copy number variation (CNV) is now recognised as an important contributor to congenital heart disease (CHD). This review surveys recent advances in the field of structural genomics, with the goal of informing both the clinical translation of findings and the anticipated future wave of whole-genome sequencing (WGS) studies. Herein, CHD refers to major malformations of the heart or great vessels present at birth. Cardiomyopathies, vasculopathies and cardiac arrhythmias are excluded. The focus is on findings from large-scale genome-wide studies of submicroscopic germline CNV, and not on chromosomal abnormalities detectable on karyotype, case reports or small case series of individual CNVs, or deletion/duplication analyses of individual genes.

## Clinical description and epidemiology of CHD

CHD is the most common class of major congenital anomalies in humans, and a major source of morbidity and paediatric mortality around the world.^1–3^ Most incidence estimates range from 4 to 10 in 1,000 live births.^1–3^ Bicuspid aortic valve, isolated aneurysm of the atrial septum, and persistent left superior vena cava are usually excluded from these incidence figures.^1^ Although most cardiac defects can be diagnosed prenatally by fetal echocardiography, many anomalies are not identified on routine prenatal ultrasound.^4–7^ Prenatal diagnosis of severe CHD can improve outcomes.^5,7–9^ Signs and symptoms are related to the type and severity of the heart defect. Many, but not all, cardiac lesions are identified in childhood. Surgical repair creates a new cardiac phenotype with unique associated late sequelae. Despite advances in early detection and surgical repair, CHD remains the leading global cause of non-infectious neonatal death.^3,9^ The vast majority of patients who survive this early period require lifelong specialised cardiac care.^3,10–13^

**Figure 1:**
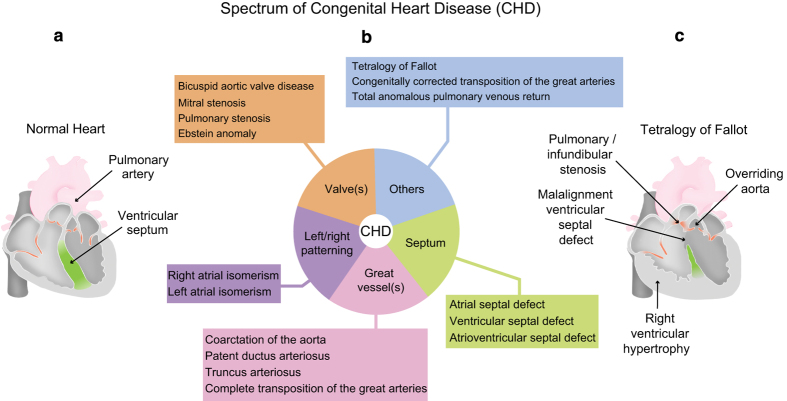
Spectrum of human CHD. CHD is an umbrella term for a range of malformations of the heart and great vessels (aorta and pulmonary arteries). There exist multiple clinically and anatomically discrete lesions, of differing incidence and severity. See text and associated references for details. (**a**) Labelled diagram of the structurally normal human heart. (**b**) Examples of some congenital cardiac lesions, based on anatomy. For a full list of congenital cardiac defects, consult a congenital cardiology textbook. Multiple congenital defects may be present within an individual. (**c**) Labelled diagram of one specific form of CHD: tetralogy of Fallot (TOF).

Beginning at 3 weeks of embryonic life, the heart is the first organ to start developing in the human embryo.^14^ An overview of the complex sequence of events that results in a well-formed heart at birth is beyond the scope of this review.^14^ Disruption of any developmental step can result in a cardiac malformation. Anatomical, functional and clinical categorisations exist for the myriad described cardiac lesions (Figure 1). Advances in imaging technology have led to improved diagnostic precision.^7^ Various constellations of multiple defects can be found together, and can result in more complex CHD, of which tetralogy of Fallot (TOF) is the most common form (Figure 1).^11,12^ CHD may occur in apparent isolation, or in conjunction with other, extra-cardiac features (syndromic CHD). Overall, individuals with CHD are at increased risk for extra-cardiac congenital anomalies and for neurodevelopmental problems.^15,16^

With advances in paediatric surgery, the prevalence of individuals living with CHD is increasing in many areas of the world.^2,3^ Adults with CHD now outnumber affected children.^17^ With increased longevity, there is more attention on long-term outcomes and causation. Evidence from family and twin studies supports a genetic aetiology for CHD.^18–21^ However, classic Mendelian inheritance patterns are usually not observed, reported recurrence risks are low, and there are low concordance rates of CHD within families.^18–21^ Non-genetic factors, e.g., teratogens such as alcohol or infections like rubella, or other comorbidities like maternal type 1 diabetes mellitus, may have a role in increasing risk for CHD and may interact with genetic predisposition.^22,23^ For these reasons CHD has long been considered an archetypal model for multifactorial inheritance.^20,24,25^ Until recently, the main molecular genetic insights into the aetiology of CHD involved large chromosomal anomalies visible on karyotype.

## Context: early studies of chromosomal imbalances in CHD

Major chromosomal anomalies including aneuploidies have been associated with CHD for over half a century.^20^ The major aneuploidies compatible with postnatal life are traditionally associated with certain types of CHD: Down syndrome/trisomy 21 (atrioventricular canal defect), Edward syndrome/trisomy 18 (ventricular septal defect or VSD, pulmonary stenosis), Patau syndrome/trisomy 13 (VSD, atrial septal defect, transposition of the great arteries or TGA), Turner syndrome/monosomy X (coarctation of the aorta, aortic stenosis, VSD), and Klinefelter syndrome/XXY (Ebstein anomaly, TOF).^26^ It is important to note however that a broad spectrum of CHD has been observed within each of these syndromes.

Although genome-wide, the resolution of karyotype is low, rarely identifying structural anomalies less than 5–10 Mb in size. Chromosomal abnormalities visible on karyotype often involve deletion or duplication of hundreds of genes, and expression of complex developmental phenotypes.^20,27^ Karyotype was thus not often helpful to identify individual genes important to cardiac morphogenesis. Over 20 years ago, a technological advance became clinically available that used targeted probe testing with fluorescence *in situ* hybridisation to provide a molecular diagnosis for selected microdeletion syndromes associated with CHD.^20^ Testing required a high clinical index of suspicion and knowledge of the targeted syndrome to allow the specific probe to be ordered.

Fluorescence *in situ* hybridisation was instrumental in revealing the presence of submicroscopic structural anomalies associated with identifiable genetic syndromes, e.g., 7p11.23 and 22q11.2 deletions, respectively, for Williams–Beuren syndrome and 22q11.2 deletion syndrome (22q11.2DS), the latter formerly known as DiGeorge/velocardiofacial syndrome.^28,29^ Studies of these recurrent, usually *de novo*, 1.5–3 Mb sized CNVs have proven useful to identify individual genes (*ELN* at the 7p11.23 locus and *TBX1* and *CRKL* at the 22q11.2 locus) that are important in cardiac morphogenesis.^28,29^ Animal models with genetically engineered deletions and point mutations of selected genes have been instrumental in these studies.^28–30^

These early findings have left the impression that most chromosomal anomalies associated with CHD: (i) arise as *de novo* mutations; (ii) usually result in multiple congenital extra-cardiac features making them readily identifiable in the newborn period; (iii) have a rather specific pattern of associated CHD per individual syndrome (e.g., supravalvular aortic stenosis in Williams–Beuren syndrome); and (iv) individually account for relatively few patients with CHD. It would take new molecular technologies to reveal otherwise.

## Genome-wide CNV in health and disease

**Figure 2:**
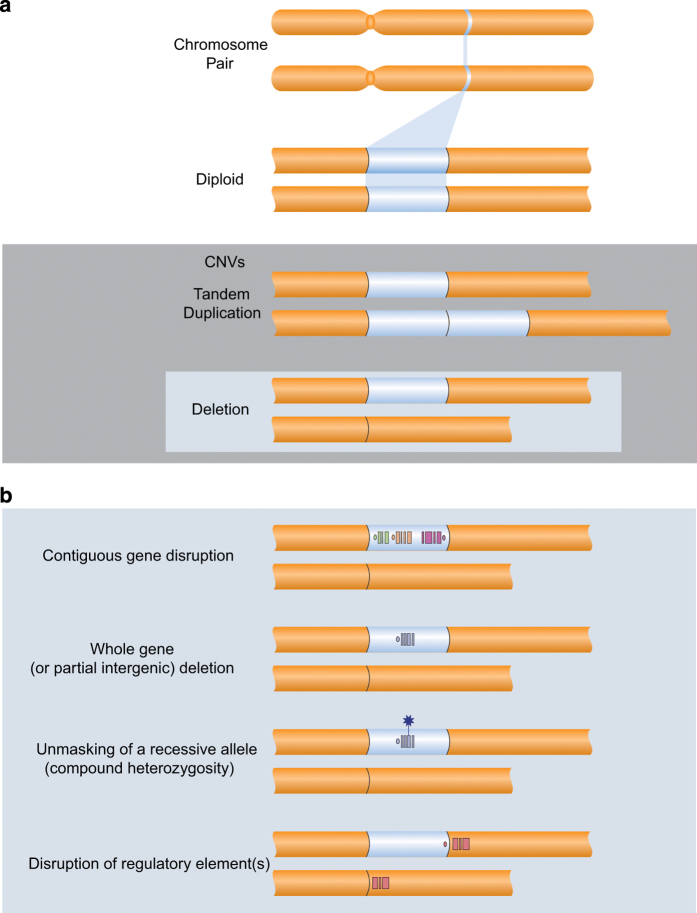
Examples of CNV and associated disease mechanisms. (**a**) Normal diploid status and two examples of CNV (a simple deletion and a tandem duplication). Not pictured are the diverse other forms of CNV, including non-contiguous insertions, higher-order copy number changes (multi-allelic CNV), and more complex rearrangements. CNVs may involve no, one or multiple genomic elements. (**b**) Selected mechanisms underlying disease effects of copy number losses (deletions). A gene is indicated by a contiguous monochromatic set of rectangles, and a regulatory element (e.g., promoter) by an oval. The definition of ‘gene’ extends beyond protein-coding genes to potentially include noncoding elements like microRNAs and long noncoding RNAs. Of note, duplications can effect change through increased copy number of a dosage sensitive gene (not pictured) or via the mechanisms depicted for deletions (e.g., via disruption at a breakpoint or partial intragenic duplication). Inspired by Figure 1 in ref. 92 and Figure 2 in ref. 33.

A breakthrough discovery in 2004 showed that structural genomic variants (CNVs) are, as a class, common in the general population.^31,32^ These include both losses (deletions) and gains (duplications) with diverse potential mechanisms of action (Figure 2).^33^ CNVs are not inherently pathological. Within any individual’s genome, there are typically both myriad common (i.e., >1–5% frequency in the general population), and one or more rare, CNVs. The mutation rate of structural rearrangements like CNVs is much higher than that of single base pair changes, and CNVs may disrupt none, one, or several genomic elements (Figure 2).^31–33^ Some ‘recurrent’ CNVs are individually rare but arise with similar breakpoints in unrelated families because of an underlying genomic architecture that predisposes to CNV. CNVs that are common may act as neutral variants or sometimes as modifiers of disease susceptibility. Rare CNVs are far more likely than common CNVs to be associated with disease, especially developmental disorders like CHD.^25,33–36^ As for virtually all genetic variants, CNVs, including rare pathogenic CNVs, are usually associated with some degree of variable expression and/or incomplete penetrance. Increasing knowledge about CNV has revolutionized thinking about differences between individual human genomes and the genetic architecture of common complex (multifactorial) disorders like CHD.^25^

## Genome-wide CNV in CHD

Over the past decade strong evidence has accumulated that genome-wide rare CNV represents a considerable source of the genetic variation that contributes to CHD susceptibility.^37–56^ These findings include both known associations such as chromosome 22q11.2 deletions, and new discoveries across the genome. Studying CNV in CHD is contributing to our understanding of cardiac morphogenesis and related disorders,^25^ and to new knowledge that is relevant for clinical practice.

### Increased overall burden of rare CNVs

A consistent finding across multiple genome-wide studies is that there is an excess burden of rare CNVs in CHD compared with control populations (Table 1).^38–40,42–55,57,58^ Differences in the definition of rarity, size cut-offs, overlap of genes and/or coding sequence (exons), array platforms and the CNV calling algorithm(s) used, other technologies, exclusions (e.g., karyotypic anomalies, 22q11.2 deletions) and CHD sample ascertainment strategies, will all affect the proportions of individuals reported to have rare CNVs. The majority of patients studied were putatively non-syndromic. Most studies excluded 22q11.2 deletions and other diagnosable syndromes (Table 1). This may obscure the fact that a relatively large proportion of individuals with CHD have 22q11.2 deletions.^29,59^ TOF is the best-studied cardiac lesion but all CHD adequately studied to date shows an excess burden of rare CNVs. Both gain and loss CNVs are involved, with certain loci recurring (see below), in addition to the 22q11.2 deletion. One attempt at a meta-analysis identified dozens of putatively associated loci.^52^ Other CNVs appear so rarely that recurrence has not yet been observed. Most studies have reported only CNVs involving autosomes; sex chromosome copy number findings may also be relevant to CHD.^60^

### Increased burden of *de novo* CNVs

Restricting to rare CNVs that have arisen as *de novo* mutations shows similar excess burden findings in CHD, even after excluding 22q11.2 deletions (Table 2). Few studies employed direct control populations; however, the general population rate of *de novo* CNVs is well studied in other cohorts and typically quoted as <2%.^47,57^ One study involving patients with TGA reported a lower *de novo* mutation rate than for other cardiac lesions.^55^ This would be consistent with offspring recurrence data for TGA that suggest fewer dominant-acting mutations for this CHD.^53^ However, the numbers of patients studied to date is too small to draw firm conclusions (Table 2). The elevated *de novo* CNV mutation rate in other severe CHD may in part explain how it is maintained in the population, despite a strong negative selective pressure and before the advent of modern paediatric surgery. Although rare *de novo* mutations are of interest and often considered to be more highly penetrant, it is important to note that most CNVs, even rare CNVs known to be pathogenic, are inherited. Moreover, recurrent CNVs that arise *de novo* in some patients are inherited from parents without CHD in others (see below), consistent with variable expression and in some cases reduced penetrance.

### 22q11.2 deletions and duplications

The role of 22q11.2 deletions in CHD was well delineated before the advent of chromosomal microarray analysis (CMA).^29^ Cumulative prevalence estimates of this recurrent 22q11.2 deletion include ~50% in interrupted aortic arch type B, ~33% in truncus arteriosus, ~15% in TOF and 5–10% in VSD.^29,59^ Appreciation for other genome-wide rare recurrent and non-recurrent CNVs in CHD more generally has served to reinforce the importance of 22q11.2 deletions as the archetypal model for continued genetic discovery and clinical translation of findings. From a molecular genetic perspective, human and animal studies are shedding light on the specific determinants of cardiac expression (Box 1). From a clinical perspective, diagnosis is helpful and changes management, including genetic counselling.^29,61,62^

General features of 22q11.2 deletions are proving to be generalisable to other CNVs associated with CHD. Although enriched for conotruncal and other anomalies, all types of CHD have been associated with 22q11.2 deletions. Severity ranges from non-viable (fetal and early pregnancy loss) to subclinical, e.g., spontaneously closing VSDs. Many patients do not have a CHD phenotype. If ascertainment is not through congenital cardiac clinics, the prevalence of readily detectable CHD may be as low as ~25–40%.^63^ Although multi-system expression is the norm over the lifetime, readily detectable congenital anomalies may not be present.^29^ The CHD may appear ‘isolated’, especially prenatally or at birth, as neurodevelopmental and neuropsychiatric features may not become apparent for years or decades.^29,61,62^ Dysmorphic features are usually subtle, and absence of ‘typical’ facial features does not affect the likelihood of the diagnosis being present or the severity of the presentation.^29^ Mortality is significantly increased,^29^ and surgical and perioperative complications in those with 22q11.2 deletions and conotruncal anomalies can be greater than other patients with the same CHD.^64^ The deletion may be inherited or *de novo*. If inherited, the parent with the deletion will often not have CHD or, if present, the same CHD or other features as the affected proband.^29^ There is a high mutation rate in the population due to the local genomic architecture: flanking segmental duplications and resulting increased risk of non-allelic homologous recombination during meiosis. The reciprocal duplication can result in similar phenotypes, including CHD.^29,65^

### 1q21.1 deletions and duplications

**Figure 3:**
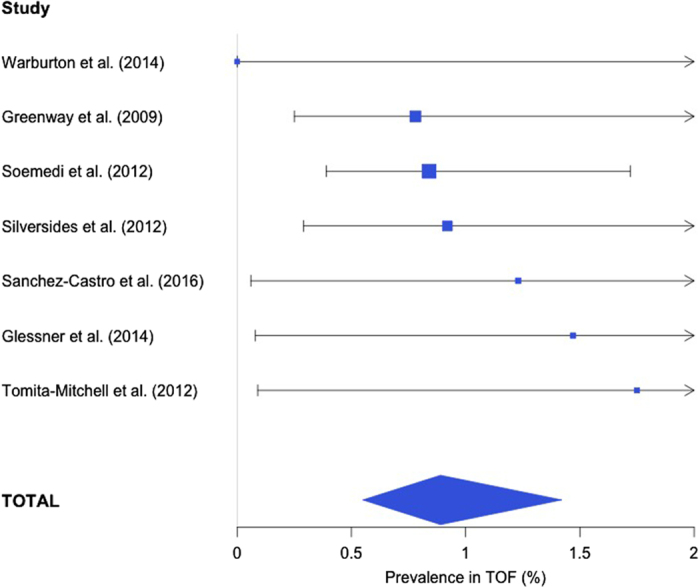
Prevalence of 1q21.1 duplications in cohorts with TOF. Box size is proportional to study size: *n*=33,^47^ *n*=510,^45^ *n*=948,^68^ *n*=433,^38^ *n*=81,^55^ *n*=68^57^ and *n*=57.^50^ The blue diamond represents the combined prevalence of 1q21.1 duplications in a total of 2130 subjects with TOF, and the diamond width corresponds to 95% confidence interval bounds. In contrast, 1q21.1 duplications are rare in control populations (see text).^67^

One of the first loci to be identified from genome-wide studies of CNV in CHD was the recurrent 1q21.1 CNV. Sometimes occurring as a deletion,^66^ but more commonly as a duplication, this is a highly replicated finding, especially in TOF where the prevalence appears to be just less than one in every 100 patients (Figure 3).^38,39,45,47,50,55,57,67,68^ As for all CNVs associated with CHD, the expression is variable, even within families, for both cardiac anomalies and other phenotypes, and is not necessarily correlated with the length of the CNV.^38,40,47,52,67,69^ 1q21.1 duplications are rare in control populations, e.g., in five out of 18,828 controls (0.027%) from three studies, where some controls were not screened for disease (reviewed in ref 67). Although it is uncertain if any single gene at this locus can be designated as truly causal, the most evidence points to the *GJA5* gene that encodes connexin 40,^38,68^ a cardiac gap junction protein expressed in the right ventricular outflow tract.^45^ Point mutations in *GJA5* can be associated with atrial fibrillation.^70^

### 8p23.1 deletions overlapping GATA4

Before the first wave of genome-wide CNV studies, the 8p deletion syndrome and sequence mutations in *GATA4* were both reported to be associated with CHD.^14,71,72^ Haploinsufficiency of *GATA4* is now one of the most frequent rare structural anomalies identified in genome-wide CNV studies of CHD.^34,39,41,47,48,50,52,58^ Unlike the typical chromosomal rearrangements at 1q21.1, 15q11.2 and 22q11.2 mediated by non-allelic homologous recombination, these 8p deletions are non-recurrent and of varying size. Haploinsufficiency of adjacent genomic elements like *SOX7* may shape the cardiac expression.^41,73^ Duplications at the 8p23.1 locus have also been reported in CHD.^48,50,58,60^ The spectrum of cardiac lesions favours septal defects, including atrioventricular canal defects,^52^ but as for other loci there is significant cardiac phenotypic heterogeneity. *GATA4* encodes an essential cardiac transcription factor that works in concert with other key regulators of cardiac morphogenesis like *NKX2-5* and *TBX5*.^14,71^

### 15q11.2 deletions

The putative association of 15q11.2 deletions with CHD illustrates the complexity imbued by incomplete penetrance, non-cardiac phenotypic expression, and ascertainment. Proximal deletions in this complex region (breakpoints BP1-BP2) on chromosome 15 are risk factors of modest effect for neurodevelopmental and neuropsychiatric disorders.^34,74–76^ A majority are inherited,^74^ typically from a parent with milder or no overt phenotypic consequences. With a nonspecific phenotype and prevalence in control populations of up to 1 in 400,^75^ 15q11.2 deletions are typically categorized as variants of uncertain significance (VUS).

Two early studies reported that individuals with this 15q11.2 deletion were moderately enriched in cohorts with diverse CHD, relative to control populations.^34,39^ However, one study had an increased burden of extra-cardiac phenotypes in cases,^34^ and the other had a low prevalence in the controls used (one in 1,538).^39^ Since then, 15q11.2 deletions have been identified infrequently in genome-wide CNV studies of CHD.^57^ Case series of individuals with 15q11.2 deletions ascertained through clinical testing for developmental phenotypes or multiple congenital anomalies report variable rates of CHD,^74–76^ influenced in part by screening with echocardiography.^74^ A notable proportion have had left-sided cardiac lesions,^39,57,74,75^ but the observed spectrum of cardiac lesions is broad. Specific susceptibility element(s) within this region remain to be determined. The typical deletion encompasses four highly conserved, non-imprinted genes (*CYFIP1*, *NIPA1*, *NIPA2* and *TUBGCP5*) with as yet no independent link to cardiac morphogenesis. Additional rare variants are common in these individuals and may act independently or in concert with the 15q11.2 deletion to increase risk for CHD,^75–77^ although in one case series there was similar prevalence of CHD in a smaller subgroup without secondary CNVs.^76^

### Pathway analyses and gene families

Interpreting genome-wide CNVs is challenging, as individual variants are rare and often involve multiple genomic elements. Also, non-knockout mutation dosage changes in most genes are uncharacterized in both humans and model organisms. Several studies have applied pathway and gene enrichment analyses of varying degrees of sophistication to rare CNV datasets to identify gene sets important to cardiac development. Although most were limited in scope, there are significant results for not only functional gene sets that would be expected (vascular development/cardiac structure)^47^ but also for more novel gene sets: WNT signalling,^39^ ‘gene neighbours’ (with shared genetic or physical interactions) of *GATA4*/*TBX5*/*NKX2-5*,^38,57^ angiogenesis,^40^ semaphorin-plexin pathways,^38^ ciliary proteome,^38,48^ TGF-beta signalling^48^ and neuron projection.^38^ The latter is notable because of the high rate of neurodevelopmental problems in patients with CHD, and similar findings from a large whole-exome sequencing study.^78^ There is also overlap with arrhythmogenic genes initially implicated in electrical/functional as opposed to structural heart abnormalities (e.g., *GJA5* and *CACNA1C*).^38,53,68,77^ CNV overlap of non-protein-coding genes like microRNAs is less well studied but may also implicate cardiac pathways.^79,80^

## Clinical genetic testing with CMA

Diagnosis of microdeletion and microduplication syndromes previously necessitated a high index of suspicion for classic clinical features that may or may not be present. CMA (also known as array comparative genomic hybridisation) is a clinical genetic test that allows for the identification of not only the individually rare CNVs underlying these established syndromes but also other emerging ‘genomic disorders’ across the genome. Initial consensus testing indications for CMA were multiple congenital anomalies and/or developmental delay/intellectual disability/autism spectrum disorder.^35^ Various outcomes are possible with CMA (Box 2).

The clinical yield of CMA in CHD has been best described in the prenatal setting. Jansen *et al.*^81^ identified 13 publications that included 1,131 cases in their meta-analysis of array comparative genomic hybridisation studies in prenatally diagnosed CHD. The incremental yield (i.e., excluding aneuploidy and 22q11.2 deletions) of clinically relevant CNVs was 7.0%; 3.4% in ‘isolated’ CHD and 9.3% in syndromic CHD.^81^ When including 22q11.2 deletions, the overall yield was 12% (about one in eight).^81^ An additional benefit of CMA was in the detection of atypical 22q11.2 deletions that are not detectable using standard probes in targeted fluorescence *in situ* hybridisation testing. VUS were identified in an additional 3.4% of CHD fetuses.^81^ Thus for pre-test counselling, one might predict a 14% chance of uncovering an anomaly on CMA that is deemed clinically reportable: 4% 22q11.2 deletion, 7% other pathogenic CNV and 3% VUS.^81^ One caveat is that pathogenic CNV included both those variants implicated in CHD, as well as ‘incidental’ findings relevant to neurodevelopment. The largest component study^82^ drove most of the significant findings in this meta-analysis. As more knowledge accumulates, original results of such genetic testing need to be re-annotated with respect to pathogenicity. Postnatal studies support a similarly high and increasing yield of pathogenic CNVs in both isolated and syndromic CHD cohorts,^83,84^ as well as a high rate of VUS.^85^ As expected, the yield is consistently higher in individuals with syndromic CHD.^51,83^

Indications for genome-wide testing are likely to expand. In practice, CMA is now often offered for apparently isolated forms of serious CHD.^84,85^ Some have advocated for the universal postnatal use of CMA in isolated CHD.^86^ In consanguineous families, single-nucleotide polymorphism arrays offer the added benefit of determining regions of loss of heterozygosity, which may then direct targeted sequencing of recessive genes implicated in cardiovascular development. The cost of testing and the current high rate of VUS are important disadvantages. Another major caveat is that most single gene disorders are not detectable with CMA. Patients, families and clinicians may mistakenly conclude on the basis of a normal CMA result that an identifiable genetic contribution to the CHD has been ruled out (Box 2). The application of CMA, while helpful for providing a molecular diagnosis for a significant minority of patients with CHD, is not a replacement for a comprehensive medical genetic evaluation.

## Recurrence risk estimation and genetic counselling

Genetic counselling in CHD has traditionally relied upon empiric recurrence risk values,^87,88^ but rare CNVs can have a significant impact in specific cases. As in other domains, classic microdeletion syndromes like 22q11.2DS are the archetypal example of clinical applicability. Other genetically diagnosable subtypes of CHD promise similar possibilities with respect to changing standard recurrence risk prediction and genetic counselling.^67,87,88^

Incorporating rare CNVs into risk prediction and counselling requires a nuanced approach. Careful phenotyping and genotyping of the parents is essential.^87^ A *de novo* CNV may be mistaken for the sole, causal variant in an individual with CHD. In one example, a subject with left-sided CHD and a *de novo* partial 1q21.1 duplication also inherited two rare CNVs overlapping candidate genes for CHD from a less severely affected father.^40^ *De novo* rare CNVs can seemingly have no, or subclinical deleterious effects. Parental germline mosaicism is also a consideration. In one recent study of rare CNV in CHD, the parents of two out of 20 affected probands with apparently *de novo* CNVs were ultimately found to have low level mosaicism in serum.^47^

Most rare CNVs are inherited. Some, such as 22q11.2 deletions, are recurrent and/or have been observed in unrelated individuals, while others are ultra-rare or unique to a particular family (private). Inheritance of a CNV from a purportedly unaffected parent can be falsely reassuring. As discussed above, variable expression of cardiac phenotypes is the norm. The effects of high but incompletely penetrant mutations on reproductive fitness in the parental generation are an important source of bias.^89,90^ A parent may also have a subclinical cardiac phenotype (e.g., bicuspid aortic valve demonstrates familial recurrence with hypoplastic left heart syndrome^91^) or a non-cardiac phenotype that may not be readily apparent. Alternatively, inheritance of any variant from an affected parent has an *a priori* likelihood of at least 50%. Co-segregation of a variant with CHD within a nuclear family (i.e., from affected parent to affected child) is thus suggestive but not specific. Two large-scale studies have considered multiplex nuclear families,^40,47^ but none to date have adjudicated CNVs within the extended family context. Counselling always needs to emphasise the potential for considerable variability in the type and severity of CHD, and in any extra-cardiac manifestations.

Prognostication in paediatric probands and concerns about sibling recurrence were previously the primary impetus for clinical genetic testing and counselling. With improved childhood survival, there is growing interest in issues germane to adults with CHD,^10–12^ including offspring recurrence.^19,88^ Irrespective of informative molecular genetic findings, surveillance during pregnancy, including fetal echocardiography, is the standard of care for offspring of both women and men with CHD. In the setting of a putative susceptibility CNV in either parent, there may be opportunities for pre-implantation genetic diagnosis or prenatal genetic diagnosis (with chorionic villus sampling or amniocentesis). The above caveats about interpreting inherited variants are relevant in this situation. Whether genetic subtyping with respect to rare CNV could also inform general obstetrical management in women with CHD is a question that requires further study.

## Clinical prognostication and prediction of extra-cardiac features

Rare CNVs are associated with a range of developmental disorders and major extra-cardiac phenotypes.^33,34,92^ Available data suggest that ‘syndromal’ patients with TOF, including those with recognisable genetic syndromes, have worse surgical outcomes and worse 10-year actuarial survival.^93,94^ Gaynor *et al.*^95^ showed that neurodevelopmental outcomes were also significantly worse in infants with CHD who had confirmed or suspected genetic syndromes. Notably, patient-specific factors were more important predictors of worse neurodevelopmental outcomes than intraoperative factors.^95^ There is a clinical impetus to better understand and predict phenotypic expression of rare CNVs across the lifespan, particularly as regards surgical and neurodevelopmental outcomes. In general, the larger and rarer the rearrangement the more easily observable the phenotypic effects.^33^ Given comparably sized rearrangements at the same locus, deletions usually have more severe phenotypic effects than duplications.^33^ Last, extra-cardiac features can appear later in life.^61,77,92,96^ For this reason, the ‘syndromic’ label is difficult to apply reliably and may be misleading, particularly in children and in the prenatal setting.

Two studies have explored the potential consequences of large rare CNVs on early outcomes, after excluding aneuploidies and 22q11.2 deletions. Carey and colleagues studied 223 patients with single-ventricle CHD and data on neurocognitive and growth outcomes at 14 months.^58^ They identified putatively pathogenic rare genic CNVs >300 kb in size in 14% (25 duplications and six deletions), including CNVs associated with genomic disorders in 13 patients. Comparing those with a deletion or duplication (CNV+) to those without such a CNV showed some subtle effects on growth associated with duplications, and worse neurocognitive outcomes on one of two measures in the small subgroup with deletions. Of note, worse neurodevelopmental outcomes are associated with large deletions in other populations without CHD.^97,98^ As anticipated, worse neurocognitive outcomes were associated with the genomic disorders associated with recurrent CNVs.^58^ Only three out of the 14 subjects examined in the CNV+ group had dysmorphic features or significant extra-cardiac phenotypes.^58^ Recently, Kim *et al.*^54^ reported that the presence of a rare genic CNV >300 kb in size was also independently associated with decreased transplant-free survival in a separate cohort of 422 children with non-syndromic CHD.

With increasing survival into adulthood, genetic factors that may inform longevity and reproduction are clinically, as well as scientifically, relevant. Late cardiac complications result in significant morbidity and mortality; however, not all patients develop adverse late outcomes (cardiac or extra-cardiac).^10–12,99^ Adults with 22q11.2DS, with and without CHD, have been studied.^89,100^ One study of reproductive fitness in adults with TOF and without 22q11.2DS showed that syndromal subjects were more likely to be childless, but failed to identify any association with the rare CNV profile.^19^ A smaller adult cohort with TGA, ascertained and studied in a similar manner, showed comparable results.^53^ A majority of deaths in adults with CHD are a consequence of cardiovascular complications, including heart failure, arrhythmias and sudden death.^10–12,99^ Whether rare CNV burden is also associated with later clinical outcomes is unknown.

## Insights into human cardiac development, and implications for discovery-based science

Several genetic mechanisms and many genes are involved in increasing susceptibility to CHD. Microarray technology for genome-wide CNV detection has greatly accelerated the pace of discovery of new candidate genes and pathways. Ongoing research to delineate copy number variable regions in the genome and measure their frequencies in the general population, at ever-higher resolutions, is key to being able to interpret results from disease populations. Efforts to catalogue CNV loci associated with CHD are valuable (see, e.g., the meta-analysis by Thorsson *et al*.^52^), but any such list requires regular updating and revision.

CNVs can narrow critical regions for CHD, including those previously implicated by large karyotypically-visible anomalies. Hundreds to thousands of protein-coding genes are expressed in the human heart or great vessels at some point in early development. The complex interplay of multiple proteins and regulatory elements may lead to relative weak spots susceptible to disruption by mutations, yet this susceptibility is balanced by the innate redundancy of these systems. Disruption of normal cardiac morphogenesis requires overcoming the robustness of genetic networks that have been fine-tuned through evolution. By virtue of their size and mutational frequency, CNVs can simultaneously disrupt multiple genes and/or regulatory elements within an individual (Figure 2), and this could be why CNV is so important to CHD causation. In addition to the examples provided above, studies involving the 1p36 deletion syndrome nicely illustrate this concept, as well as the associated challenges in ascribing causality to specific genes.^101–105^ Only a minority of rare CNVs however will result in complex multi-system developmental phenotypes and/or *in utero* or early neonatal mortality.

The considerable variability in the type and severity of CHD associated with individual recurrent CNVs provides further support for, and new insights into, the genetically and functionally interdependent pathways that govern cardiogenesis. There appears to be significant shared underlying genetic susceptibility to lesions considered anatomically discrete, akin to the genetically related spectrum of seemingly disparate neurodevelopmental disorders.^34,92^ Combining clinically distinct CHD (e.g., multiple conotruncal defects) can increase power for rare variant identification and analyses.^39,53,57^ Common and rare genetic modifiers of expression remain to be discovered (Box 1).

## Future considerations

Individually rare CNVs—gains and losses, inherited and *de novo*—are collectively important genetic factors contributing to abnormal heart and great vessel development in humans. All rare variants sufficiently studied to date are characterised by some degree of variable cardiac and extra-cardiac expression. Many also display reduced penetrance. Estimating these parameters is challenging, and can be confounded by ascertainment bias and reproductive fitness effects.^19,67,89,90^ The individual rarity of non-recurrent CNVs has meant that there is often insufficient evidence (be it epidemiology, cell biology or model organism-based) to conclude causality. Large human sample sizes including the wealth of data generated from clinical use of CMA, high-throughput model organism screening,^106^ and targeted mutagenesis using CRISPR/Cas9 technology will all help in this endeavour.

Epistatic interactions are not well studied in human CHD. Our ability to provide increasingly personalised counselling will be determined by our understanding of modifiers of expression. Elegant experiments in mice with heterozygous *Nkx2-5* mutations demonstrate the layers of complexity that we can expect to encounter even in individuals with pathogenic single-gene mutations.^107,108^ For CNV, genome-wide molecular studies in cohorts with 22q11.2 deletions provide the template for studying other recurrent variants (Box 1). For example, an immediate next step for many CNVs would be to carefully define the breakpoints and study the intact allele (Figure 2). The success of this approach is best illustrated by distal 1q21.1 deletions causing TAR syndrome.^109^

In our experience, the primary hope of many families and clinicians is that prenatal or early postnatal genetic, e.g., CMA, testing will help in predicting neurodevelopmental outcomes. This has not been the focus of most studies reviewed herein. However, research on CNV in CHD has been occurring in parallel with studies of developmental delay/intellectual disability, autism spectrum disorder and schizophrenia.^33–36^ Our understanding of genome-wide CNV, in terms of both general mechanisms (Figure 2) and specific loci, has been heavily influenced by neurodevelopmental disorders. A component of neurocognitive outcomes in individuals with CHD is genetically determined, and this can be dissected using the same approaches as studies of developmental disorders more generally. The expectation is that the frequency of VUS will decrease and the helpfulness of pathogenic CNV diagnoses will improve as more data accrue. The escalating uptake of methods that facilitate prenatal detection of CNV, including non-invasive prenatal testing that now permits screening for selected recurrent microdeletions,^110^ will further stoke demands for reliable prognostication and genetic counselling.

There is an impetus to consider in a similar fashion to CNV and structural variation the contribution of rare sequence-based changes to the genetic architecture of CHD. New genetic technologies have proved useful in advancing our understanding of CHD aetiopathogenesis, and increasing clinical diagnostic yield. Recently, whole-exome sequencing has begun to facilitate novel gene discovery.^78,111^ In the two largest whole-exome sequencing study to date, Zaidi *et al.* and Homsy *et al.* demonstrated enrichment of *de novo* point mutations in individuals with severe CHD and identified multiple new candidate genes.^78,111^ Interpretation of rare sequence variants will be informed by genes and pathways identified through rare CNV. WGS is the next logical step in this progression. The ability to comprehensively assess sequence and structural changes in coding and noncoding sequence genome-wide within an individual with CHD will undoubtedly identify new mechanisms that govern human cardiac morphogenesis. WGS offers the promise of improved resolution with respect to structural variation compared with CMA. The path forward involves the application of WGS on a large-scale. This will need to be married with careful phenotyping, family studies, and other hallmarks of excellence in genetic discovery study design. Personal WGS as a single universal genetic test in CHD that is cost-effective and of broadly applicable clinical utility is a highly anticipated but future consideration.^112^

## Figures and Tables

**Table 1:** Case–control studies of genome-wide rare CNV burden in CHD^a^

*Study*	*Cases (n)*	*CHD type*	*Controls (n)*	*Significant case–control CNV burden findings*	*22q11.2 deletions excluded or absent*
Zhao *et al.*^43^	100	Various^b^	65	Increased proportion of subjects with rare CNVs >100 kb in size (39.0 vs 21.5%)	Yes^c^
Costain *et al.*^53^	101^d^	TGA	415^e^	Increased proportion of subjects with rare CNVs >500 kb in size (10.1 vs 4.6%)	Yes
Carey *et al.*^58^	223	Single ventricle	270	Increased proportion of subjects with rare genic CNVs >300 kb in size (13.9 vs 4.4%)	Yes
Fakhro *et al.*^48^	262	HTX	991	Increased proportion of subjects with rare genic CNVs (14.5 vs 7.4%)	Yes
Kim *et al.*^54^	422	Various^b^	500	Increased proportion of subjects with rare genic CNVs >300 kb in size (12.1 vs 5.0%)	Yes
Silversides *et al.*^38^	433^d^	TOF	416^e^	Increased proportion of subjects with rare CNVs >500 kb in size (9.1 vs 5.1%)^f^	Yes
Soemedi *et al.*^39^	2,256	Various^b^	841	Increased proportion of subjects with rare genic loss CNVs (7.8 vs 4.4%)	Yes

Abbreviations: ASD, atrial septal defect; CHD, congenital heart disease; CNVs, copy number variations; HLHS, hypoplastic left heart syndrome; HTX, heterotaxy; PDA, patent ductus arteriosus; TGA, transposition of the great arteries; TOF, tetralogy of Fallot; VSD, ventricular septal defect.

a Minimum *n*=100 case subjects. Glessner *et al.*^57^ reported an increased burden of *de novo* CNVs in CHD cases relative to controls, but multiple numerical inconsistencies in their report resulted in its exclusion from this table.

b Zhao *et al*.^43^: ASD (*n*=58), VSD (*n*=22), PDA (*n*=15), TOF (*n*=2), Ebstein anomaly (*n*=2), and tricuspid incompetence (*n*=1); Kim *et al*.^54^: HLHS (*n*=130), TOF (*n*=64), TGA (*n*=34), VSD (*n*=40), VSD/coA (*n*=19), single ventricle (*n*=30) and other (*n*=105); Soemedi *et al*.^39^: the four largest of the 29 categories were TOF (*n*=808), ASD (*n*=293), TGA (*n*=165) and VSD (*n*=163).

c Included one atypical 22q11.2 deletion overlapping *CRKL*.

d Only subjects of European ancestry were considered in burden analyses.

e All rare CNVs in cases and controls were adjudicated for rarity by comparing to those in additional population-based controls: *n*=2357,^38^ *n*=10 113.^53^

f Also within-TOF finding of more exonic losses in the syndromic subgroup.

**Table 2:** Studies of genome-wide *de novo* CNV rate in CHD (22q11.2 deletions excluded)^a^

*Study*	*Recruitment site(s)*	*Case trios (n)*	*CHD type*	*Array type(s)*	*De novo rate per subject*^b^	
Hitz *et al.*^40^	Canada (QC)	53	Left-sided	Affymetrix Human Genome-Wide SNP Array 6.0 (Santa Clara, CA, USA)	6/53	11.3%
Xie *et al.*^42^	South Central China	82	PA	Illumina 660W-Quad & Omni1-Quad BeadChips (San Diego, CA, USA)	12/78	15.4%
Greenway *et al.*^45^	USA (Boston), Brazil	114	TOF	Affymetrix Human Genome-Wide SNP Array 6.0	9/112	8.0%
Warburton *et al.*^47^	USA (NY)	223	CNT, HLHS	NimbleGen CGH HD2 (Madison, WI, USA)	20/213	9.4%
Soemedi *et al.*^39^	UK, Germany, Belgium, Australia	283	TOF	Illumina 660W-Quad	13/283	4.6%
Sanchez-Castro *et al.*^55^	France	316	CoA	Agilent 2*400K (Santa Clara, CA, USA, custom-designed)	3/76	3.9%
			TOF		5/81	6.2%
			TGA		0/159	0.0%
Glessner *et al.*^57^	USA (various)	538	Various^c^	Illumina Omni-1.0 and 2.5M^d^	47/534	8.8%

Abbreviations: CHD, congenital heart disease; CNT, conotruncal anomalies; CNVs, copy number variations; CoA, coarctation of the aorta; HLHS, hypoplastic left heart syndrome; HTX, heterotaxy; PA, pulmonary atresia; TGA, transposition of the great arteries; TOF, tetralogy of Fallot; VSD, ventricular septal defect.

a Minimum *n*=50 trios.

b Proportion of unrelated case subjects with at least one *de novo* CNV, after excluding individuals with 22q11.2 deletions.

c Left-ventricular outflow lesions>CNT>>heterotaxy>other (exact numbers cannot be determined from data provided).

d Also employed whole-exome sequencing for characterisation of CNV.

## Genetic studies of 22q11.2 deletion syndrome (22q11.2DS) as a guide for understanding variable expression

The 22q11.2 deletion alone cannot account for the variable expression of the congenital cardiac phenotype in 22q11.2DS.^29^ Efforts are underway to dissect the layered genetic architecture that gives rise to this variability. Progress to date offers a blueprint for studying pathways from CNV genotype to CHD phenotype more generally.

* Within the 22q11.2 region:*

• Length of the deletion and specific breakpoints
  - There is little to no discernible association of severity of the phenotype, including the CHD, with the length of the deletion.^113^
  - Haploinsufficiency of multiple genes within the region appears to be important, including *TBX1*,^30^ *CRKL*^114^ and *DGCR8*.^115^
  - Sequence variation in *TBX1* on the intact 22q11.2 allele does not have a major role in cardiac expression.^116^
• Animal models of the full deletion and of haploinsufficiency of specific deleted genes
  - Haploinsufficiency of multiple genes within the region appears to be important, including *TBX1*,^30^ *CRKL*^114^ and *DGCR8*.^115^
  - Sequence variation in *TBX1* on the intact 22q11.2 allele does not have a major role in cardiac expression.^116^
• Variation on the intact allele
  - Sequence variation in *TBX1* on the intact 22q11.2 allele does not have a major role in cardiac expression.^116^

*Outside the 22q11.2 region (genome-wide):*

• Family studies
  - Family history data support a heritable susceptibility to CHD in families of probands with *de novo* 22q11.2 deletions and CHD.^117,118^
  - A common duplication of *SLC2A3* was significantly enriched in individuals with 22q11.2 deletions and CHD, implicating this CNV as a possible genetic modifier in a small proportion of subjects.^113^
  - Preliminary data suggest that rare CNVs outside the 22q11.2 region may overlap genes that modify risk for CHD in a small proportion of subjects.^119^
  - Whole-exome sequencing implicated rare mutations in histone modification genes as possibly influencing risk for CHD in the presence of the 22q11.2 deletion.^120^
• Common genome-wide variation
  - A common duplication of *SLC2A3* was significantly enriched in individuals with 22q11.2 deletions and CHD, implicating this CNV as a possible genetic modifier in a small proportion of subjects.^113^
  - Preliminary data suggest that rare CNVs outside the 22q11.2 region may overlap genes that modify risk for CHD in a small proportion of subjects.^119^
  - Whole-exome sequencing implicated rare mutations in histone modification genes as possibly influencing risk for CHD in the presence of the 22q11.2 deletion.^120^
• Additional rare CNV
  - Preliminary data suggest that rare CNVs outside the 22q11.2 region may overlap genes that modify risk for CHD in a small proportion of subjects.^119^
  - Whole-exome sequencing implicated rare mutations in histone modification genes as possibly influencing risk for CHD in the presence of the 22q11.2 deletion.^120^
• Additional rare sequence mutations
  - Whole-exome sequencing implicated rare mutations in histone modification genes as possibly influencing risk for CHD in the presence of the 22q11.2 deletion.^120^

## Outcomes of clinical genetic testing with CMA

There are four general outcomes of CMA: (1) no abnormality (normal array); (2) pathogenic variant; (3) VUS; and, rarely, (4) secondary or incidental finding. Categories 2–4 may co-occur. Many laboratories follow the American College of Medical Genetics and Genomics (ACMG) guidelines for interpretation and reporting of postnatal constitutional CNVs.^121^

1. *Normal array:* In the case of a normal array, the cause of the individual’s CHD remains unexplained. Counselling about a negative result includes the fact that this is the likeliest outcome. This normal result does not preclude the likelihood that other genetic testing may be clinically available now or in the future that could help to resolve the aetiology of the CHD. CMA will not detect sequence mutations, or chromosomal imbalances below a certain resolution.
2. Example: A normal array in a patient with truncus arteriosus and a loss-of-function mutation in *NKX2-5*.
3. *Pathogenic variant:* Discovery of a pathogenic CNV that is a known risk factor for CHD will prompt a recommendation for a referral to medical genetics and/or a sub specialty clinic for further specific assessment and counselling. Parental testing where possible is usually recommended. In some cases, parental studies will uncover the same CNV or perhaps a balanced chromosome rearrangement that confers a significant recurrence risk for having other children with a pathogenic CNV.
4. Example: A typical 1q21.1 duplication in a patient with TOF.^67^
5. *VUS:* VUS are those CNVs that are not yet reliably characterised as either benign or pathogenic. Subcategories exist: ‘VUS; likely pathogenic’, ‘VUS; likely benign’ and ‘VUS (no sub-classification)’.^121^ Genetic assessment and counselling may be recommended about what is known now, and/or in the future should new information arise. VUS, especially those deemed ‘likely pathogenic’, are often a source of future pathogenic variants.
6. Example: A duplication overlapping *CACNA1C* and a typical 15q11.2 deletion in a patient with TGA.^77^
7. *Secondary finding:* There is the possibility of finding a variant associated with a genetic disorder not believed to be related to the primary reason for referral. In these rare instances, the genetic disorder may be associated with an adult onset condition.
8. Example: Haploinsufficiency of the familial breast and ovarian cancer gene *BRCA2* in a patient with TOF.^96^
